# Therapeutics of the Throat

**Published:** 1889-01

**Authors:** James B. Bell


### THERAPEUTICS OF THE THROAT.

### DIOSCOREA.

THROAT in general. *Dryness, soreness, smarting* and *burning* in the whole throat. Belching of gas, but the throat is so dry it stops the gas. Pain extending to both ears. Constant desire to swallow, but it causes nausea and shuddering.

Sharp, aching pain in both parotid glands. Constricted feeling in throat, as from something tight about the neck, makes breathing difficult.

MOUTH.—Bitter, clammy, dry, but no thirst.

LARYNX.—Constant tickling in the larynx, causing cough. Tickling in the larynx and bronchia causing a hacking cough.

COMMENTS.—The best time to cure "a cold" is in the beginning. If we can select the right remedy when the first smarting in the fauces appears, we can prevent the later development of coryza and cough which will otherwise follow with most patients.

The importance of Diosc. consists in its resemblance to this first stage of many colds. We do not get to know its modalities in this connection, but will have to compare it with Æsculus h., Berb., Cistus, Euph., Merc., N-mos., Phell., Phyt., Sabad, Sang., Seneg., Sinapis, Tellur., and others.

### INDIUM.

THROAT.—Uvula greatly enlarged. Back part of pharynx covered with thick, yellow mucus, very hard to dislodge.

Left tonsil swollen, pain and difficulty in swallowing. Throat sore on the right side. Dryness, throbbing, stinging, soreness, swallowing painful.

Throat—In the evening, and morning—*eating* and *drinking cold water*.

Tickling in the throat, inducing continued hawking.

Destructive ulceration of the uvula, soft palate, and tonsils.

LIPS.—Cold sores. Cracks in the corners.

COMMENTS.—Indium is indicated in rather sub-acute catarrhal sore throats, and possibly some specific tertiary ulcerations, after the usual abuse of Mercury. For post-pharyngeal catarrh, with a *tough, leathery streak of yellow mucus* down the back of the throat, it suits very well. In the relief from eating and drinking, it resembles Æsculus, Benz-ac., Cistus, Lach.

JAMES B. BELL.

